# High-throughput analysis of the RNA-induced silencing complex in myotonic dystrophy type 1 patients identifies the dysregulation of miR-29c and its target ASB2

**DOI:** 10.1038/s41419-018-0769-5

**Published:** 2018-06-28

**Authors:** Marisa Cappella, Alessandra Perfetti, Beatrice Cardinali, Jose Manuel Garcia-Manteiga, Matteo Carrara, Claudia Provenzano, Paola Fuschi, Rosanna Cardani, Laura Valentina Renna, Giovanni Meola, Germana Falcone, Fabio Martelli

**Affiliations:** 1 Institute of Cell Biology and Neurobiology, National Research Council-Monterotondo, Rome, Italy; 2 Molecular Cardiology Laboratory, IRCCS-Policlinico San Donato, San Donato Milanese, Milan, Italy; 3 Center for Translational Genomics and BioInformatics, IRCCS San Raffaele Scientific Institute, Milan, Italy; 4 Laboratory of Muscle Histopathology and Molecular Biology, IRCCS-Policlinico San Donato, San Donato Milanese, Milan, Italy; 5 Department of Neurology, IRCCS-Policlinico San Donato, San Donato Milanese, Milan, Italy; 6 Department of Biomedical Sciences for Health, University of Milan, Milan, Italy

## Abstract

Myotonic dystrophy type 1 (DM1) is a multi-systemic disorder caused by abnormally expanded stretches of CTG DNA triplets in the *DMPK* gene, leading to mutated-transcript RNA-toxicity. MicroRNAs (miRNAs) are short non-coding RNAs that, after maturation, are loaded onto the RISC effector complex that destabilizes target mRNAs and represses their translation. In DM1 muscle biopsies not only the expression, but also the intracellular localization of specific miRNAs is disrupted, leading to the dysregulation of the relevant mRNA targets. To investigate the functional alterations of the miRNA/target interactions in DM1, we analyzed by RNA-sequencing the RISC-associated RNAs in skeletal muscle biopsies derived from DM1 patients and matched controls. The mRNAs found deregulated in DM1 biopsies were involved in pathways and functions relevant for the disease, such as energetic metabolism, calcium signaling, muscle contraction and p53-dependent apoptosis. Bioinformatic analysis of the miRNA/mRNA interactions based on the RISC enrichment profiles, identified 24 miRNA/mRNA correlations. Following validation in 21 independent samples, we focused on the couple miR-29c/ASB2 because of the role of miR-29c in fibrosis (a feature of late-stage DM1 patients) and of ASB2 in the regulation of muscle mass. Luciferase reporter assay confirmed the direct interaction between miR-29c and ASB2. Moreover, decreased miR-29c and increased ASB2 levels were verified also in immortalized myogenic cells and primary fibroblasts, derived from biopsies of DM1 patients and controls. CRISPR/Cas9-mediated deletion of CTG expansions rescued normal miR-29c and ASB2 levels, indicating a direct link between the mutant repeats and the miRNA/target expression. In conclusion, functionally relevant miRNA/mRNA interactions were identified in skeletal muscles of DM1 patients, highlighting the dysfunction of miR-29c and ASB2.

## Introduction

DM1 (OMIM #160900) is a chronic, slowly progressing multi-systemic disease, with symptoms that include loss of muscle strength, myotonia, excessive fatigue, cardiac conduction defects, cataracts, insulin resistance and, in the most severe forms, cognitive impairment^1–3^. DM1 is caused by a dynamic expansion of CTG repeats, ranging from 50 to several thousands, in the 3′ untranslated region of the *dystrophia myotonica protein kinase* (*DMPK)* gene^4^. Characteristic molecular features of the disease have been associated with a toxic RNA gain of function of the CUG expansions. Expanded CUG repeats have been demonstrated to be toxic per se in several cell types and animal models^5–7^, disrupting pre-mRNA alternative splicing^8^. Mutant CUG repeats accumulate into distinctive foci within muscle nuclei and lead to dysregulation of RNA-binding proteins such as MBNL1 and CELF1. Both MBNL1 and CELF1 regulate critical alternative splicing transitions during heart and skeletal muscle development, which are dysregulated in DM1^8–11^.

Adding complexity to the molecular pathology of DM1, several other disease mechanisms have been recently found, including microRNA (miRNA) dysregulation^12–16^. miRNAs are short non-coding RNAs, which regulate gene expression by decreasing their target mRNA levels, or by blocking their translation^17–19^. miRNAs are generated by longer precursors and mature miRNAs are the product of the sequential action of Drosha and Dicer endonucleases. miRNA/target interaction is mediated by the RNA-Induced Silencing Complex (RISC), of which AGO2 is an obligatory component. The levels of a subset of miRNAs, both muscle specific (miR-206, miR-1) and not (miR-335, miR-29b, miR-29c, and miR-33), have been found to be deregulated in DM1^12–16^. For miR-1 and miR-206, contrasting data have been reported, probably depending on the low numerosity of DM1 patients studied and the disease severity, as well as the type of skeletal muscle analyzed. Importantly, we also found that intracellular distribution of miR-1, miR-133b and miR-206 was severely altered^15^. This is likely to be functionally important, since the expression of at least some of the corresponding targets was also modified.

In a DM1 cell model, it has been shown that transcripts containing long CUG-repeat hairpins are substrates of the ribonuclease Dicer and that the fragments generated can act as endogenous siRNAs, triggering a downstream silencing effect^20^. However, it is still unknown whether CUG-repeated short sequences are functionally active in vivo. Finally, *DMPK* transcripts containing expanded CUGs may also act as molecular sponges for miRNAs with CAG repeats in their seed regions^21^.

Thus, while it seems clear that miRNA pathway is disrupted in DM1, the functional implications of this dysregulation require further investigation. To identify “functional” miRNAs, actually engaged in mRNA/target inhibitions relevant for DM1 disease mechanisms, we analyzed RISC-associated RNAs in muscle biopsies derived from DM1 patients compared to healthy individuals.

## Results

### Sequencing of the RNAs associated to the RISC complex of DM1 skeletal muscles

**Fig. 1:**
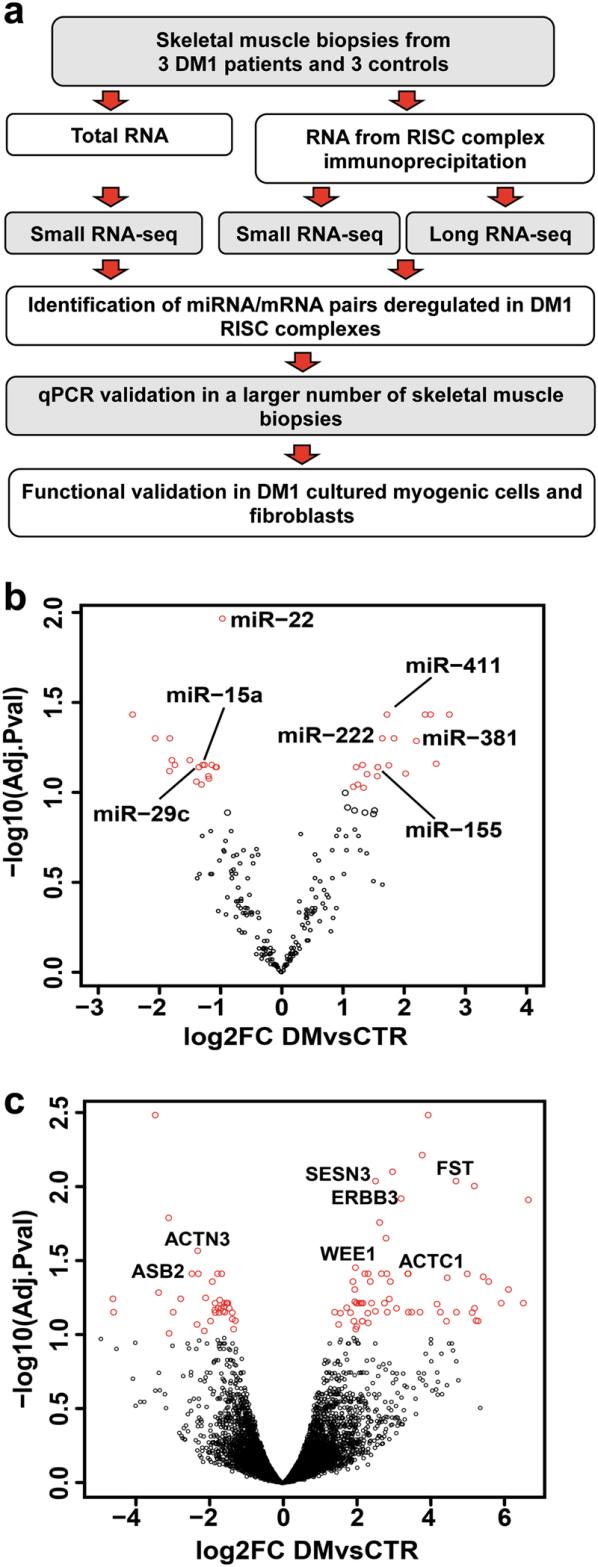
Experimental strategy and RISC-associated miRNAs and mRNAs. **a** Experimental plan outline. **b**, **c** Volcano plot of differentially modulated miRNAs and mRNAs, revealed by RISC-associated RNA-sequencing. miRNAs (**b**) and mRNAs (**c**), differentially modulated in RISC complex of DM1 patients compared to CTRs. Red dots represent miRNAs and mRNAs displaying statistically significant differences (FDR < 0.1; DM1, *n* = 3; CTR, *n* = 3). miRNAs with mRNA targets differentially modulated in the RISC, and validated mRNAs are identified by name in **b** and **c**, respectively

In order to characterize the deregulation of the RNAs associated to the RISC complex in the skeletal muscle of DM1 patients, RISC complexes were immunopurified and both miRNAs and mRNAs were analyzed by RNA-sequencing (RNA-Seq). A schematic representation of the experimental plan is shown in Fig. 1a.

Triplicate *biceps brachii* biopsies were harvested from 3 DM1 patients and from three individuals not affected by neuromuscular disorders. One biopsy was used for histological evaluation, one for total RNA extraction and one for RISC immunoprecipitation (RISC-IP). Clinical features of both DM1 patients and controls (CTRs) are summarized in Supplementary Tables S1 and S2. Histological analysis of DM1 biopsies displayed typical disease hallmarks such as centrally nucleated and atrophic myofibers, and nuclear clumps, in the absence of overt fibrosis, as previously described (Supplementary Figure S1 ^22^).

RISC-IPs were performed with antibodies to Ago2, a core component of the RISC^17, 18^ and the associated RNAs were extracted. As control for effective Ago2/RNA co-immunoprecipitation, an aliquot of the extracted RNA was analyzed by qPCR for the enrichment of small RNAs (miR-1 and miR-221), as well as of long RNAs (*CDKN1B* and *FOS* mRNAs), compared to IP performed with control antibodies (Supplementary Figure S2). As expected, U6 small nuclear 1 (*RNU6-1*) that does not associate to the RISC, displayed no enrichment.

RNA derived from the RISC-IP samples was sequenced measuring both small and long RNAs, allowing the identification of 201 miRNAs as well as of 12,976 between mRNAs and lncRNAs. As reference, small RNAs were sequenced from total RNAs derived from the same patients and CTRs used for RISC-IPs.

Data from bioinformatic analysis identified miRNAs and mRNAs that were differentially enriched or depleted in DM1 RISCs compared to RISCs derived from CTR biopsies. Specifically, 11 and 37 miRNAs were found selecting miRNAs for adjusted *p*-values ≤ 0.05 and 0.10, respectively (Fig. 1b and Supplementary Table S3). Of note, most miRNAs differentially modulated in the RISC of DM1 patients displayed similar trends, but failed to reach statistical significance, in total RNA counterparts (Supplementary Table S4), confirming the additional insight provided by the analysis of RISC-associated RNAs. Only two miRNAs behaved differently in total RNA-Seq: miR-1301 was not detected and miR-182 displayed very similar levels in DM1 and CTR total RNAs.

When RISC-associated mRNAs were analyzed, 35 and 103 mRNAs were identified as differentially enriched or depleted between DM1 and CTR for adjusted *p*-values of ≤0.05 and 0.10, respectively (Fig. 1c and Supplementary Table S5).

Next, we investigated the biological functions associated to the RISC-mRNAs differentially modulated in DM1 patients. To this aim, enriched biological processes and pathways were identified by ClueGO^23^. Supplementary Figure 3 shows enrichments in terms related to energetic metabolism, calcium signaling, muscle contraction, and p53-dependent apoptosis. Many of these functions have been shown to be deregulated in DM1^1–3^, suggesting an involvement of the RISC function in the disease mechanisms.

### CUG-containing RNA oligomers are undetectable in the RISC complex of DM1 skeletal muscles

It has been shown in a DM1 cell model that transcripts containing long repeat hairpins are substrates of the ribonuclease Dicer, yielding fragments that can act as siRNAs^20^. Thus, we tested whether CUG-oligomers were enriched in the RISC complexes derived from skeletal muscle biopsies of DM1 patients. It was found that CUG-oligomers were not detectable in the dataset obtained by sequencing the small RNAs associated to the RISC in DM1 patients. However, these triplet-containing RNAs were absent also in the dataset obtained from the sequencing of total small RNAs, suggesting a low representation of the CUG repeats in the libraries or a low sequencing efficiency.

Given the technical shortcomings of the RNA-sequencing technique, we attempted to measure by qPCR whether CUG-containing oligomers were enriched in the RISC of DM1 skeletal muscles. To this aim, total RNA was poly-adenylated and reverse transcribed with a poly(T) adapter into cDNAs, followed by qPCR amplification (Supplementary Figure S4a)^24^. As expected, CUG-containing RNAs were readily detectable in total RNAs extracted from DM1 muscles. However, the opposite was true for RISC-IP derived RNAs, indicating a low incorporation efficiency of CUG-containing RNAs in the RISC of DM1 skeletal muscles (Supplementary Figure S4b, c).

### Identification of miRNA/target mRNA pairs

To perform a comprehensive analysis of the miRNA/mRNA interactions based on the RISC enrichment profiles, we took advantage of the miRTrail webserver^25^. To increase stringency, only miRNAs and mRNAs displaying an adjusted *p*-value of 0.05 were considered for target identification.

Both positive and negative miRNA/mRNA correlations were considered, in keeping with the dual modality of miRNA function: translational control and mRNA degradation^18, 19^. Indeed, increased or decreased levels of a miRNA should lead to concordant increased or decreased target mRNA recruitment to the RISC for those miRNA/mRNA couples where the miRNA inhibitory role is mainly at the translational level. However, for those miRNA/mRNA pairs leading to efficient mRNA destabilization, increased or decreased miRNA incorporation in the RISC can lead to counter-regulation of the target levels and, consequently, its depletion or enrichment in the RISC, respectively. Table 1 shows the miRNA/mRNA pairs identified.

**Table 1:**
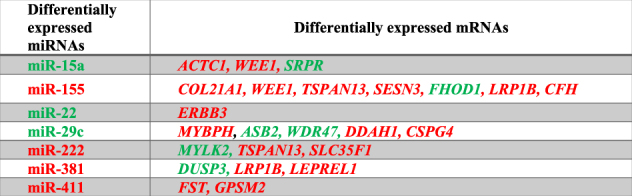
miRNA/target mRNA couples enriched (in red) or depleted (in green) in the RISC of DM1 patients

**Fig. 2:**
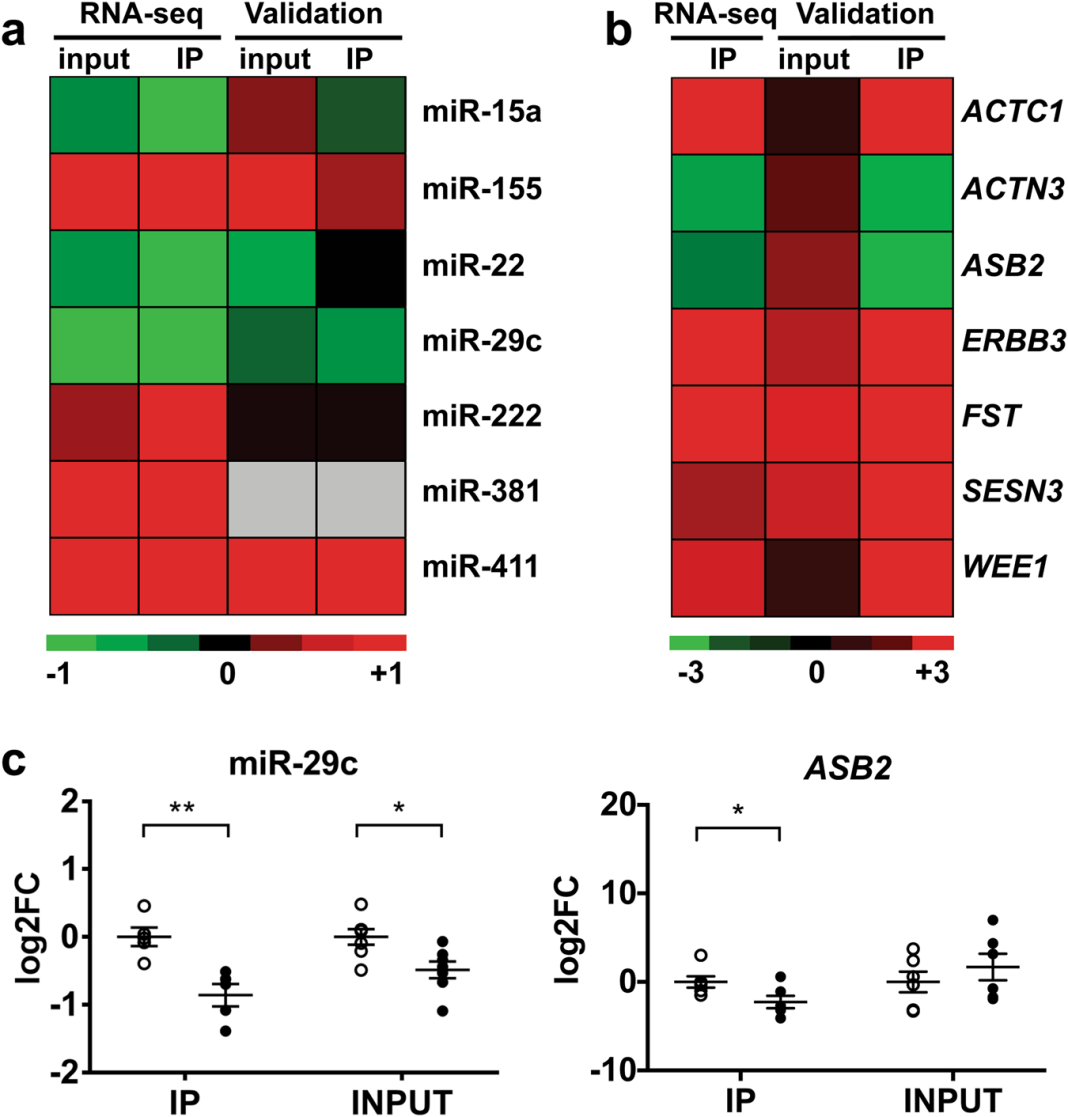
Modulation of miRNAs and mRNAs in skeletal muscle tissue. **a**, **b** Comparison between RNA-Seq results (DM1, *n* = 3; CTR, *n* = 3) and qPCR validation (DM1, *n* ≥ 5; CTR ≥ 5) in RISC-IP samples of DM1 compared to CTR subjects. Data are represented in Log2 scale, (−ΔΔCt), referred to CTRs. Upregulated and downregulated genes are indicated in red and in green, respectively. **a** miRNAs; miR-381 was not detectable by qPCR in both input and IP RNAs; **b** mRNAs; mRNA RNA-Seq of input RNA was not performed. **c** Dot plots of miR-29c and *ASB2* mRNA validation experiments. RISC-IP associated RNAs (IP) and INPUT RNAs obtained from biopsies of DM1 patients vs healthy subjects (CTR) were analyzed by qPCR and normalized to miR-181a and RPL23 mRNA, respectively. Values are indicated as −1*ΔΔCt fold changes (log2FC). Average and error bars are also indicated (CTR *n* ≥ 5; DM1 *n* ≥ 5; *P < 0.05; **P < 0.01). White circles: CTR; black circles: DM1

To validate these findings, new DM1 patients and CTRs were recruited and biopsies analyzed (Supplementary Tables S1 and S2). Given the low RNA amount that is co-immunoprecipitated with the RISC, miRNAs, and target mRNAs, selected on the basis of their relevance to disease-related pathways, were tested by qPCR only in a sample subset. However, each RNA was assayed in at least 10 samples (5 DM1 patients and 5 CTRs). Data are shown in Fig. 2a and b as heatmaps and in Fig. 2c and d and Supplementary Figure S5 as dot plots. A global agreement between RNA-Seq and qPCR data was observed, although statistical significance was not reached in all cases, possibly due to patient variability and low numerosity. miR-381 was undetectable by qPCR in RISC-IPs and could not be tested. *ACTN3* (*Alpha-actinin 3, Skeletal muscle*) was not targeted by the dysregulated miRNAs and was included as a muscle-specific control. Eventually, qPCR validation was obtained for 2 miRNAs (miR-29c and miR-411) and for 6 mRNAs: *SESN3* (*Sestrin 3*), *ACTC1* (*Actin, Alpha, Cardiac muscle I*), *FST* (*Follistatin*), *WEE1* (*WEE1 G2 Checkpoint kinase*), *ERBB3* (*Erb-B2 Receptor Tyrosine kinase 3*), and *ASB2 (Ankyrin Repeat and SOCS Box Containing 2*). Interestingly, qPCR analysis of input and RISC-associated mRNAs and miRNAs revealed a different or even opposite modulation in some cases, supporting the relevance of RISC-associated RNA analysis, compared to analysis of total RNA, to identify functionally relevant miRNA/mRNA target pairs.

### miR-29c and ASB2 deregulation in DM1 cell models

Given the role of miR-29c and ASB2 in skeletal muscle hypertrophy^26^ and in fibrosis^27^, we decided to study this miRNA/target couple in more detail.

To this aim, we took advantage of DM1 and CTR myogenic cell lines that we recently generated from patient-derived dermal fibroblasts^28^. These cells exhibited the DM1-typical ribonuclear aggregates containing CUG repeats (Supplementary Figure S6a); upon induction that triggers MyoD translocation to the nucleus, both CTR and DM1 cells differentiated efficiently (Supplementary Figure S6b).

**Fig. 3:**
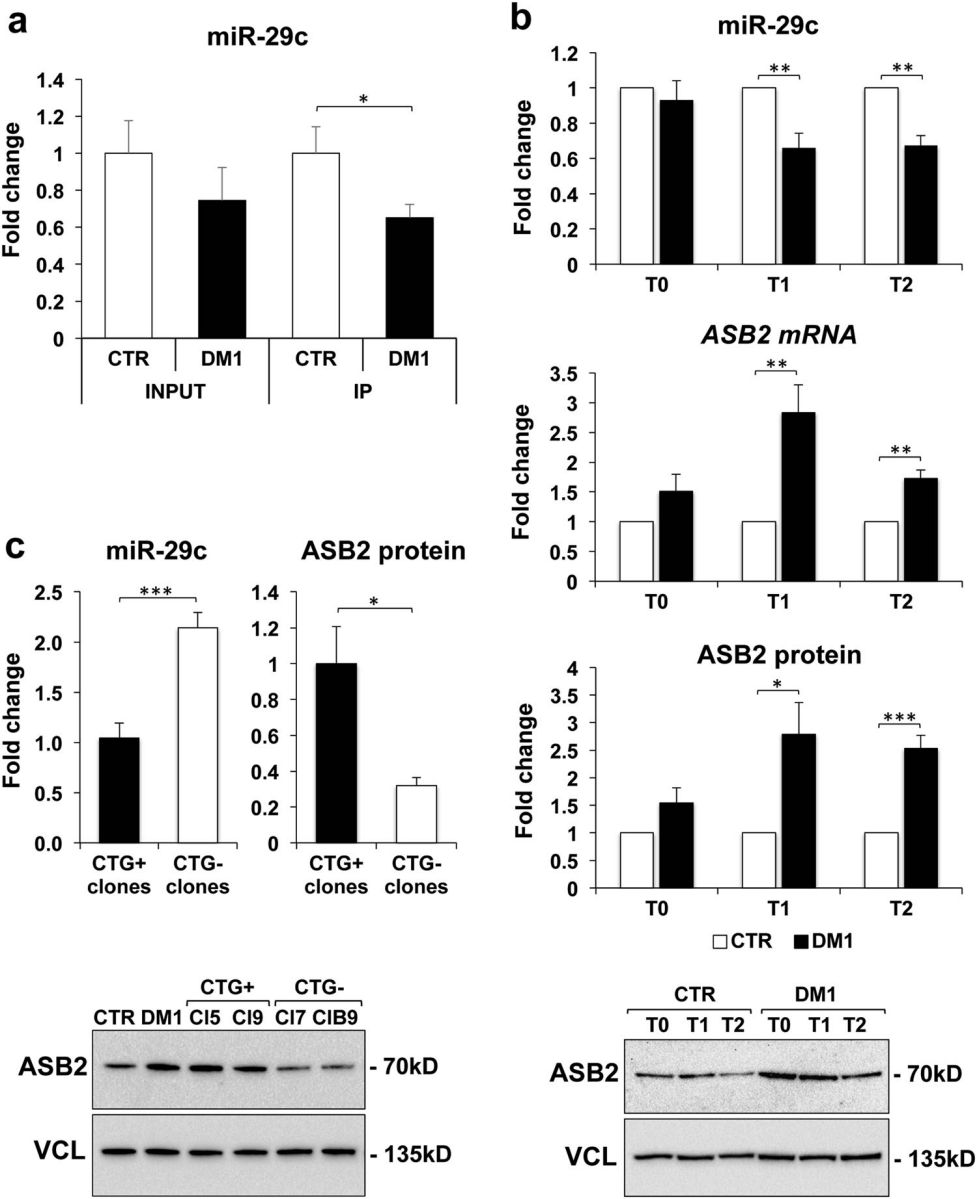
Expression of miR-29c and ASB2 in myogenic cells. **a** qPCR analysis of miR-29c in RISC complexes derived from two CTR and two DM1 cell lines allowed to differentiate for 5 days. Levels of miR-29c in both INPUT and IP were normalized on miR-181a levels, and shown as fold change of DM1 vs CTR, referred as 1 (*n* = 6; *P < 0.05). **b** Expression of miR29c and ASB2 in CTR and DM1 undifferentiated myogenic cells (T0) and myogenic cells induced to differentiate for 3 and 5 days (T1 and T2, respectively). Expression of miR-29c and *ASB2* mRNA was analyzed by qPCR (top and middle charts), normalized for expression of miR-16 and *RPL23*, respectively, while ASB2 protein expression was measured by western blot (bottom chart and gel image of a representative experiment), normalized to vinculin (VCL) expression. DM1 values were first normalized to CTR, referred as 1, for each experiment and then the average value and standard error of DM1 fold change was calculated (*n* ≥ 4; *P < 0.05; **P < 0.01; ***P < 0.001). **c** Expression of miR-29c and ASB2 protein in CRISPR/Cas9 treated clones, either maintaining CTG expansions (CTG+, average values of 2 clones) or with full deletion of repeat expansions (CTG−, average values of 2 clones), induced to differentiate for 2 days. Values were normalized for expression of miR-16 and vinculin, respectively, and shown as fold change of CTG− clones relative to CTG+ clones referred as 1. (*n* ≥ 3; *P < 0.05; ***P < 0.001). A representative western blot analysis of ASB2 protein in CRISPR-Cas9-edited clones and in untreated DM1 and CTR cells is shown

qPCR analysis of RISC-associated miR-29c in CTR and DM1 differentiated myotubes, confirmed a significant miRNA downregulation in DM1 samples (Fig. 3a), in agreement with the results obtained in skeletal muscle biopsies. In order to validate *ASB2* as a target of miR-29c, we investigated whether there was a correlation between *ASB2* and miR-29c expression both in CTR and DM1 cell models. Interestingly, analysis of miR29c and its predicted target protein ASB2 during myoblast differentiation, showed an inverse correlation between miR-29c accumulation and ASB2 transcript/protein levels in DM1 compared to CTR cells at all timepoints (Fig. 3b).

Next, we tested whether there was a direct link between miR-29c/ASB2 deregulation and CTG-repeat expansion. To this aim, we assayed DM1 myogenic cell clones in which the toxic mutant CTG-repeats were removed by CRISPR/Cas9 gene editing system^28^. These clones were negative for ribonuclear foci, exhibited normal splicing, differentiated upon MyoD induction, and formed myotubes, similarly to CTR myoblasts^28^. A significant rescue of both miR-29c and ASB2 expression was observed in these clones, compared to non-edited clones retaining full expansion (Fig. 3c).

Along with myofibers, another major cell component of skeletal muscles are fibroblasts. Indeed, fibrosis is a disease hallmark in end-stage DM1 patients (Supplementary Figure S7)^1–3^. Thus, primary fibroblasts were derived from both DM1 and CTR skeletal muscle samples and the cells cultured in vitro.

As expected, these cells were negative for desmin and positive for α-smooth muscle actin and vimentin (Supplementary Figure S8). In keeping with skeletal muscle biopsies, miR-29c was decreased and ASB2 mRNA was increased in DM1 muscle fibroblasts (Supplementary Figure S9).

Therefore, in vitro experiments confirmed that ASB2 mRNA/protein were upregulated in DM1 cells whereas miR-29c was down-modulated.

### miR-29c targets ASB2 directly

**Fig. 4:**
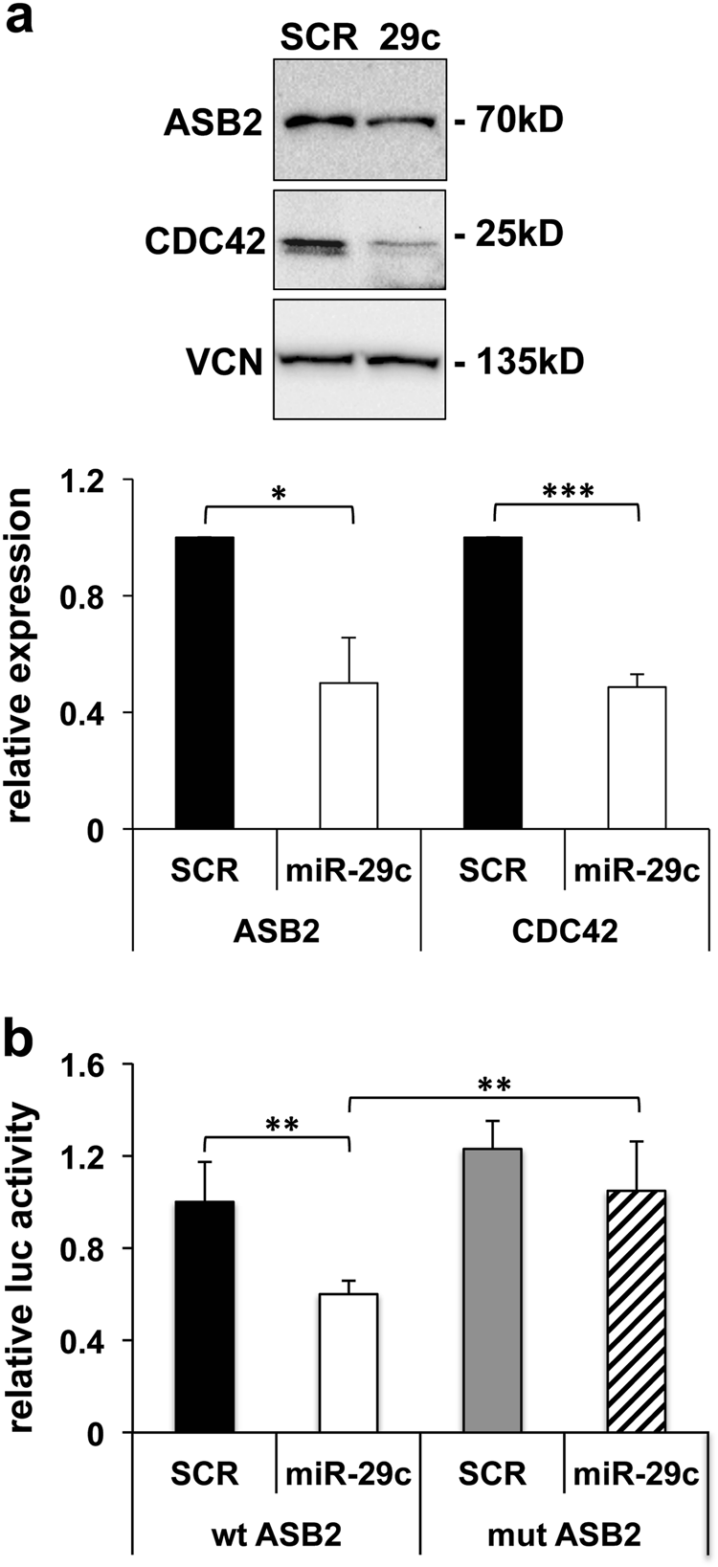
Validation of ASB2 as a direct target of miR-29c. **a** Undifferentiated myogenic cells were transfected with miR-29c- or scramble (SCR)-expression vectors and the levels of ASB2 and CDC42 were analyzed by western blotting. A representative blot is shown. Vinculin (VCN) is shown as loading control. The histogram shows the quantitation of the expression levels of ASB2 and CDC42 normalized to vinculin in cells expressing miR-29c vs cells expressing scramble, referred as 1 (*n* = 3; *P < 0.05; ***P < 0.001). **b** HEK-293 cells were transfected with empty Firefly luciferase reporter vector (pMIR-REPORT) or derived constructs containing either an intact miR-29c-binding site (wt), or a mutated miR-29c-binding site (mut) in *ASB2* 3′UTR target region. Each plasmid was co-transfected with a plasmid encoding renilla luciferase along with miR-29c- or scramble (SCR)-expression vectors. Activities of each pMIR-REPORT construct were normalized first to Renilla luciferase and then to empty vector activities. Luciferase (luc) activity in cells transfected with miR-29c is shown relative to activity in cells transfected with scramble RNA, referred as 1 (*n* ≥ 11; **P < 0.01)

To provide further evidence of a direct miR-29c/ASB2 regulation, we tested whether miR-29c overexpression decreased ASB2 protein levels. Thus, fibroblasts were transfected with a miR-29c expressing plasmid and ASB2 was assayed. Figure 4a shows that, indeed, miR-29c expression decreased ASB2 protein levels as much as those of CDC42, a bona fide miR-29c target^29^.

To confirm that miR-29c targets *ASB2* directly, miR-29c pairing-sites or their mutated versions and the immediately surrounding sequences contained in *ASB2* were cloned downstream of a luciferase open reading frame. The luciferase activity of these constructs was evaluated following the overexpression of either miR-29c or a scramble sequence in HEK-293 cells. Figure 4b shows that miR-29c inhibited luciferase expression from the reporter constructs containing an intact miR-29c binding site, whereas this effect was prevented by the mutation of the seed-complementary nucleotides. These data demonstrate that miR-29c inhibits ASB2 expression directly.

## Discussion

Different studies highlighted miRNA dysregulation in DM1^12–16^. However, the functional consequences of this dysregulation are far from being elucidated. In this investigation, we analyzed both miRNAs and mRNAs associated to the RISC in muscle biopsies derived from DM1 patients and CTRs. By this approach, we identified, in an unbiased manner, miRNA/target interactions that were altered in DM1. Moreover, the analysis of patient-derived skeletal muscle biopsies facilitated the identification of pathways and functions potentially relevant for DM1 disease mechanisms. As a confirmation of the additional insight provided by this approach, most miRNA dysregulations identified in the RISC did not reach statistical significance when total RNA was analyzed. It is worth noting that, due to the complexity of the RISC-IP/RNA-Seq approach, we had to start from a limited number of biopsies, possibly missing some modulated miRNAs. While this work gives an important contribute to our understanding of miRNA role in DM1, future profiling of a higher patient number might allow to identify other miRNA/mRNA couples displaying higher variability.

The analysis of RISC-associated RNAs was also important to understand the functional relevance of CUG-oligomers originating from the Dicer activity on *DMPK-*mutated transcripts^20^. Indeed, while small CUG-containing RNAs were readily detectable in total RNAs extracted from DM1 muscles, we did not find evidence of their association to the RISC, questioning their proposed role on target inhibition by RISC-mediated RNAi^20^.

The identification of deregulated mRNAs associated to the RISC was also very informative. As discussed below, this facilitated the identification of relevant targets of the dysregulated miRNAs. Moreover, a broad picture of the transcriptomic alterations in DM1 was provided. It is likely that many of the identified deregulations were not due only to altered recruitment by the miRNAs, but also to the combined action of different mechanisms, such as altered transcription. However, this analysis allowed the identification of pathways and functions relevant for the disease, such as energetic metabolism, calcium signaling, muscle contraction, and p53-dependent apoptosis^1–3^.

Bioinformatic analysis of the miRNA/mRNA interactions based on the RISC enrichment profiles, identified 24 miRNA/mRNA correlations. It is worth noting that, while miR-29c^15, 16^ and miR-381^30^ alterations were identified before, all the other miRNAs identified had escaped previous studies based on total RNA analysis.

The miRNAs deregulated in DM1 and their targets are involved in muscle damage and disease. Indeed, miR-15a targets *ACTC1*, a characterizing gene for the enrichment of the “muscle contraction” categories among the RISC-associated mRNAs.

miR-155 impairs myogenesis^31, 32^; moreover, its targets *TSPAN13* and *LRP1B* are also targeted by miR-222 and miR-381, respectively, suggesting a potential cooperative action between these miRNAs.

miR-22 is enriched in both cardiac- and skeletal muscles, is upregulated during myocyte differentiation and is a critical regulator of cardiomyocyte hypertrophy^33^. Moreover, *ERBB3*, a target of miR-22, was the characterizing gene that started the enrichment of the calcineurin-NFAT signaling cascade among the RISC-associated mRNAs^34^.

We previously identified miR-222 as a critical regulator of skeletal muscle cell differentiation and maturation^35, 36^. Moreover, miR-222 target *Myosin Light Chain Kinase 2* (*MLCK2*) was the characterizing gene for the enrichment of the “skeletal muscle development” and of the “muscle contraction” categories among the RISC-associated mRNAs.

MiR-381 was previously identified by our group as increased in DM2^30^. MiR-411 targets *FST*, a physiological myostatin inhibitor promoting skeletal muscle hypertrophy^37^.

We have characterized in more detail the dysregulation of the miR-29c/ASB2 couple and shown that ASB2 mRNA is a direct target of miR-29c. Moreover the deletion by genome editing of the CTG amplifications in a DM1 cell model rescued miR-29c/ASB2 dysregulation, linking miR-29c regulation to the presence of CTG amplifications. Further studies are needed to investigate the molecular mechanisms inducing miR-29c downregulation in DM1. Of note, a miRNA-sequestration mechanism by CUG repeats has been proposed, although no evidence was provided for miR-29c specifically^21^.

miR-29-family miRNAs display a crucial role in the regulation of extracellular matrix genes and in fibrosis^27^, suggesting that a similar role might be played also in DM1. Accordingly, analysis of cell cultures derived from DM1 patients allowed to identify decreased miR-29c levels in both myogenic and fibroblastic cells. While overt fibrosis was not particularly present in the analyzed proximal muscles of low-mid grade patients, fibrosis is a DM1 disease hallmark in higher grade patients, especially in distal muscles, such as the *tibialis anterior*^1–3^. Further studies are necessary, but it is tempting to speculate that decreased miR-29c levels might be prognostic of fibrosis becoming more evident as the disease progresses.

ASB2 is a subunit of a multimeric E3 ubiquitin-ligase complex that mediates the degradation of cytoskeletal components, such as filamin B^38^ and desmin^39^. Thus, ASB2 regulates cell spreading^40^ and migration^41^.

Type 1 fiber atrophy is a histological trait characterizing DM1 disease in the muscle^22^. Interestingly, ASB2 has been shown to negatively regulate muscle mass and its repression is necessary for FST-induced muscle hypertrophy^26^.

Additionally, a specific ASB2 isoform, ASB2beta, is important for muscle differentiation through the targeting of filamin B to destruction by the proteasome^38^. It is worth noting, however, that the identified miR-29c binding site is in the 3′UTR of the ASB2 gene that is common to alpha- and beta-ASB2 isoforms.

In conclusion, we found that the characterization of the RNAs associated to the RISC complex allowed to identify combinations of miRNAs and their target mRNAs altered in DM1 patients and likely relevant for the disease.

## Methods

### Human cells and tissues

This study was authorized by the Institutional Ethics Committee (miRNADM of 23.06.2015) and was conducted in accordance with the principles expressed in the Declaration of Helsinki, the institutional regulation, and Italian laws and guidelines. All human specimens were obtained after receiving written informed consent. Clinical diagnosis of DM patients was based upon International Consortium for Myotonic Dystrophies guidelines^42^, and genetic analysis was carried out to confirm DM diagnosis, as previously described^43^. CTR biopsies were derived from subjects admitted with suspected neuromuscular disorder of undetermined nature. CTR biopsies did not show overt signs of muscle pathology upon on histological and immune-histochemical examination and patients scored negative upon genetic testing. Patient clinical features are summarized in Supplementary Table S1. Human muscle biopsies from *biceps brachii* were harvested under sterile conditions and snap frozen in liquid nitrogen or immediately processed for histological evaluation and cell culture.

### Cell cultures and transfections

All cells were incubated under a 5% CO_2_ atmosphere at 37°C. HEK-293 (ATCC) cells were grown in DMEM (Sigma-Aldrich, St. Louis, MO) supplemented with 10% FBS (EuroClone, Milan, IT). Primary skeletal muscle fibroblasts were derived from *biceps brachii* biopsies by negative selection for CD56, a myoblast marker^44^, as previously described^45^. The cell suspensions were negatively selected using magnetic microbeads (MACS, Milteny Biotec, Bergisch Gladbach, DE) conjugated with anti-CD56 antibodies, in order to separate myogenic from primary fibroblasts. CD56-negative cells were then cultured in DMEM (Sigma-Aldrich, St. Louis, MO), 15 % FBS (EuroClone, Milan, IT).

DM1 and CTR myogenic cell lines and CUG repeats-edited clones were previously described^28^. Briefly, myogenic cell lines were derived from human fibroblasts immortalized and converted to myoblasts by transduction of the *TERT* and inducible *MYOD1* genes. Cells were derived from two DM1 patients carrying 290 (DM1-A) and 520 (DM1-B) CTG amplifications at diagnosis, respectively, and from two CTR subjects (CTR-A and CTR-B). Clones maintaining CTG expansions (CTG+ clones 5 and 9) or with full deletion of repeat expansions (CTG− clones 7 and B9) were generated using CRISPR/Cas9 genome editing from DM1-A myogenic cell line.

DM1 and CTR myogenic cells were propagated in DMEM without phenol red (GIBCO, ThermoFisher Scientific, Waltham, MA) supplemented with 15% FBS (EuroClone; Milan, IT). In these conditions, no myogenic markers were expressed. To induce myogenic differentiation, cells were grown to confluency on dishes coated with 0.5% gelatin (Sigma-Aldrich, St. Louis, MO) and proliferation medium was replaced with differentiation medium consisting of DMEM without phenol red supplemented with 10 μg/mL insulin (Sigma-Aldrich, St. Louis, MO), 100 μg/mL transferrin (Gibco, ThermoFisher Scientific, Waltham, MA), and 10^−7^ M β-estradiol (Sigma-Aldrich, St. Louis, MO).

For miRNA overexpression experiments, undifferentiated myogenic cells were electroporated with miExpress™ Precursor miRNA Expression plasmid miR-29c (HmiR0025-MR04, GeneCopoeia Inc., Rockville, MD) and miRNA Control Vector (CmiR0001-MR04, GeneCopoeia Inc., Rockville, MD) according to the manufacturer’s instructions (1 µg of DNA for 10^6^ cells) (Amaxa Basic Nucleofector Kit VP1-1002, Lonza, Basel, Switzerland). After electroporation cells were allowed to recover in growth medium for 3 days and then processed for further analysis.

### RISC immunoprecipitation

Two lysis protocols were used in muscle biopsies and cultured cells: (1) skeletal muscle biopsies were cut into small pieces in RIP lysis buffer (Magna RIP Kit, Merck-Millipore, Burlington, MA) and homogenized by TissueLyser (Qiagen, Hilden, Germany); (2) Cultured cells were lysed with the following buffer: 150 mM KCl, 25 mM TrisHCl pH 7.4, 5 mM EDTA, 0.5% NP-40, 5 mM DTT, 1 mM PMSF, protease and RNAse inhibitors. For both lysates, homogenates were quantified for protein content, snap frozen in liquid nitrogen and stored at −80°C until use. For both muscle tissue and cells, 50 μg of lysate were used for total RNA analysis (INPUT RNA) and 1 mg was immunoprecipitated with anti-Ago2 or control mouse IgG antibodies (Abs) (RIPAb+ Ago2, Merck-Millipore, Burlington, MA), using 5 μg of antibody for 1 mg of lysate. Immunoprecipitation was performed using the Magna RIP Kit (Merck-Millipore, Burlington, MA) following the manufacturer’s instructions. IgG-bound RNA (IP RNA) was extracted from magnetic beads using TRIzol reagent (Invitrogen, ThermoFisher Scientific, Waltham, MA).

### RNA-Seq library preparation and analysis

RNA-Seq was performed on small RNAs and long RNAs derived from RISC-IP samples, while for total RNA only small RNAs were sequenced. For long RNAs, rRNA-depletion and library preparation were performed following manufacturer’s instructions for the Nugen OVATION RNA-Seq System V2 and Nugen OVATION Ultralow Library Systems, while for small RNA analysis, extraction and library preparation were performed following manufacturer’s instructions for the TruSeq Small RNA Library Preparation Kit, but with a higher number of PCR cycles (*n* = 17), to compensate for the low amount of RNA analyzed. An Illumina HiSeq 2500 was used to perform a paired-end sequencing with 100 bp long reads. The average estimated insert size for mRNA and lncRNA was 150 bp. The sequencing protocol for small RNAs included pooling of samples in four pools containing both IP small RNAs and total small RNAs. After trimming of sequencing adapters and low quality sequences (Trimmomatic), the quality was checked with FastQC. BWA aligner was used to align small RNA to the hg19 human genome and STAR for longRNA reads. Coverage was measured using HTseq-count^46^ against the hg19 human transcripts (Gencode) for long RNAs or the miRBase (v19) for miRNAs. Linear Models for Microarray Analysis (LIMMA) with the voom function was used to find differentially overrepresented miRNAs^47^. RNAseq datasets for long RNAs RISC-IP, small RNA RISC-IP and small RNA total RNA are publicly available on Gene Expression Omnibus (GEO) under the accession number GSE108592.

### miRNA predicted targets and gene ontology analysis

miRNA predicted targets were determined using miRTrail tool (http://mirtrail.bioinf.uni-sb.de/upload.php)^25^ amongst the differentially enriched/depleted miRNAs and mRNAs in RISC complexes.

Enrichment in GO terms from the Biological Process tree and KEGG pathways was calculated using ClueGO^23^ app for Cytoscape^48^, set to report terms with a corrected *p*-value < 0.05.

### Gene expression analysis by qPCR

Total and immunoprecipitated RNAs were extracted with TRIzol reagent (Invitrogen, ThermoFisher Scientific, Waltham, MA). For mRNA analysis, retro-transcription was performed with the SuperScript IV Reverse Transcriptase (ThermoFisher Scientific, Waltham, MA) using oligo (dT) and random primers for cultured myoblasts and with Ovation qPCR system (Nugen, San Carlo, CA) for human biopsies. Specific primers for mRNA analysis by qPCR are shown in Supplementary Table 2. Power SYBR Green PCR master mix (Applied Biosystems, ThermoFisher Scientific, Waltham, MA) was used to analyze mRNAs. Results were normalized with respect to *RPL23* expression. miRNA levels were determined using the TaqMan MicroRNA Assays (Applied Biosystems, ThermoFisher Scientific, Waltham, MA) and samples were normalized to miR-181a expression in experiments from muscle biopsies, since this miRNA was equally expressed in DM1 and CTR RNAs according to RNAseq. Total miR-29c levels in DM1 and CTR cell cultures were instead normalized to similarly expressed miR-16, as previously reported^15^ and as assessed in set up experiments (not shown). For both miRNAs and mRNAs, qPCR was performed using an Applied Biosystem StepOnePlus or 7500 Fast Real-Time PCR System; relative expression was calculated using the comparative Ct method (2−ΔΔCt).

### qPCR analysis of small repeated RNAs

Total RNAs and immunoprecipitated RNAs were poly-adenylated in vitro using the Poly(A) Tailing Kit (Applied Biosystems, ThermoFisher Scientific, Waltham, MA) following the manufacturer’s instructions. Samples were then annealed with an oligo(dT)-adaptor primer (Supplementary Table S6) prior to retro-transcription, performed with the SuperScript III Reverse Transcriptase (Invitrogen, ThermoFisher Scientific, Waltham, MA). Specific primers recognizing CTG triplets and the adaptor (Supplementary Table S6) allowed the amplification of specific products by PCR, performed using the GoTaq Flexi DNA polymerase (Promega, Madison, WI). PCR products were sequenced using the reverse adaptor primer. Primer synthesis and sequencing were performed by Eurofins Scientific (Luxembourg, Luxembourg).

### RNA FISH and immunofluorescence stainings

Cells were fixed with 2% formaldehyde and subjected to FISH using a (CAG)6 probe labelled with Texas Red at the 5′ end (IDT, Coralville, IA) in combination with MBNL1 immunofluorescence staining, as described previously^28, 49^. For staining of fast myosin heavy chain (MHC), differentiated cultures were fixed with 80% acetone and washed thoroughly before incubation with primary (MF20, obtained by D. Fischman, Cornell Medical College, New York, NY) and secondary Ab (Alexa Fluor 488 goat anti-mouse Ab, ThermoFisher Scientific, Waltham, MA). Nuclei were visualized with Hoechst 33258 dye. The samples were examined with an Olympus AX70 immunofluorescence microscope. Images were recorded on an Olympus XM10 camera and processed using the Olympus CellSens Standard 1.8.1 software.

Skeletal muscle fibroblasts were fixed in 4% paraformaldehyde, permeabilized with 0.4% Triton in PBS (GIBCO, ThermoFisher Scientific, Waltham, MA) and incubated with Normal Goat Serum (NGS, Dako, Santa Clara, CA). Subsequently, cells were incubated with primary Abs for Desmin (clone D33, Dako, Santa Clara, CA), Smooth Muscle Actin (clone 1A4, Sigma-Aldrich, Saint Louise, MO) and Vimentin (clone V9, Sigma-Aldrich, Saint Louise, MO) and Alexa Fluor 488 secondary Ab (Molecular Probes, Eugene, OR). Nuclei were counterstained with Dapi (Molecular Probes, Eugene, OR). All Abs were diluted in PBS containing 2% Bovine Serum Albumin (BSA, Sigma-Aldrich, Saint Louise, MO). Random images were acquired using an Olympus IX51 microscope equipped with an Olympus TH4-200 camera and Cell F 2.8 software (Olympus software imaging solution). All images were evaluated in blind by at least two investigators.

### Western blot analysis

Cells were lysed in RIPA buffer (140 mM NaCl, 3 mM MgCl_2_, 1 mM EDTA, and 15 mM HEPES, pH 7.2, 0.5% sodium deoxycholate, 1% NP-40, and 0.1% SDS) supplemented with a cocktail of protease inhibitors (Roche, Sigma-Aldrich, St. Louis, MO). Western blots were carried out using horseradish-peroxidase-conjugated goat anti-rabbit and anti-mouse Abs (Santa Cruz Biotechnology, Santa Cruz, CA) and revealed with a chemiluminescence detection system by Cyanagen (Bologna, Italy). Rabbit polyclonal Abs to ASB2 and to CDC42 were purchased from Novus Biologicals (Littleton, CO) and from Santa Cruz Biotechnology (Santa Cruz, CA), respectively. Abs to vinculin (clone VIN-11-5) and β-actin (clone AC-74) were purchased from Sigma-Aldrich (St. Louis, MO). Imaging and quantitation of the bands were carried out by the ChemiDoc XRS Western Blot Imaging System using the ImageLab 4.0 software (Bio-Rad, Hercules, CA).

### Luciferase reporter assay

To construct the ASB2-wt and ASB2-mut luciferase reporter plasmids, 80nt synthetic oligos (Supplementary Table S6) encompassing the predicted miR-29c target sites (wt) or the same mutated sequence (mut, G → T, C → G and C → A) of ASB2 3′UTR were synthesized (Eurofins Genomics, Milan, IT) and cloned into the SpeI and HindIII restriction sites of the pMIR-REPORT Luciferase vector (Ambion, ThermoFisher Scientific, Waltham, MA). For the expression of luciferase reporter constructs, 7 × 10^3^ HEK-293 cells were plated on 96-multiwell plates and transfected, using the FuGENE® HD transfection reagent (Promega; Madison, WI), with 7.5 ng of pMIR-Report plasmids and 45 ng of miR-29c or scramble sequence expressing plasmids, along with 7.5 ng of pRL plasmid coding for renilla luciferase (accession number AF025844, Promega, Madison, WI) to normalize for transfection efficiency. The luciferase activity was measured using Dual-Glo Luciferase Assay System kit (Promega, Madison, WI) and the luminescence was recorded by VarioskanLux (ThermoFisher Scientific, Waltham, MA) luminometer. Each experiment was performed in quadruplicate.

### Statistical analysis

GraphPad Prism v.4.03 software (GraphPad Software Inc., San Diego, CA) was used for statistical analysis. Continuous variables were analyzed by Student’s *t*-test, Welch’s *t*-test, Mann–Whitney test or ANOVA, as opportune. All statistical tests were performed two-sided and a *P* < 0.05 was considered as statistically significant. Continuous variables were expressed as mean ± standard error of the mean (SEM).

## Electronic supplementary material

Table S1

Table S2

Table S3

Table S4

Table S5

Table S6

Supplementary figures
