# Supplementary material for: High-throughput analysis of the RNA-induced silencing complex in myotonic dystrophy type 1 patients identifies the dysregulation of miR-29c and its target ASB2
Source: Cell Death Dis. 2018 Jun 28;9(7):729. doi: 10.1038/s41419-018-0769-5 (PMC6023919; doi:10.1038/s41419-018-0769-5)
Supplement: Supplementary file 7 — Supplementary figures [file 41419_2018_769_MOESM7_ESM.pdf]

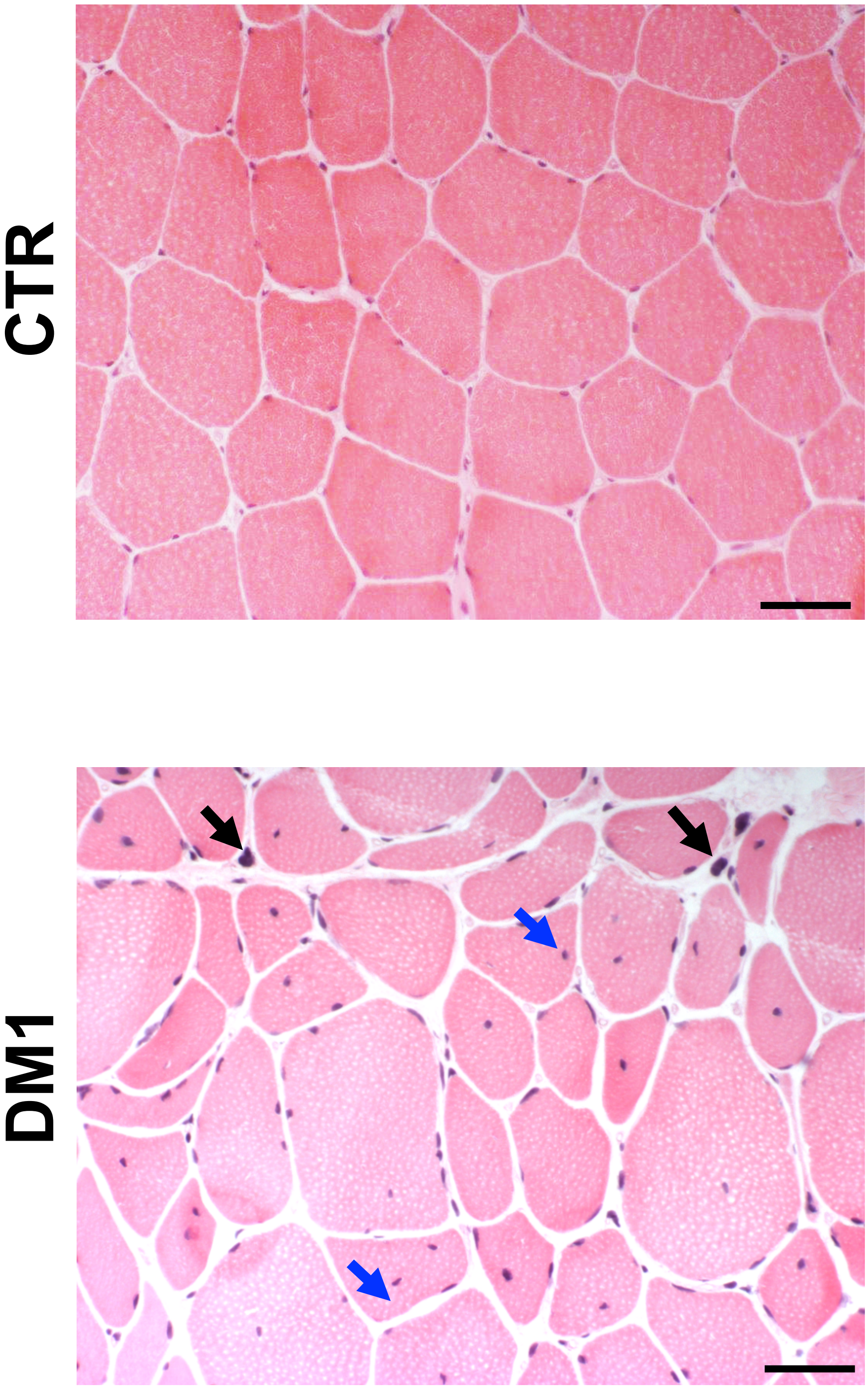

Figure S1. Histo-pathological features of *biceps brachii* muscle biopsies. Representative hematoxylin and eosin staining on frozen muscle sections of DM1 patients and CTR. High variation in fibers size, nuclear clumps (black arrows), central nuclei (blue arrows) and atrophic fibers (asterisks) are typically present in DM1, but not in control biopsies. Scale bar: 50 µm.

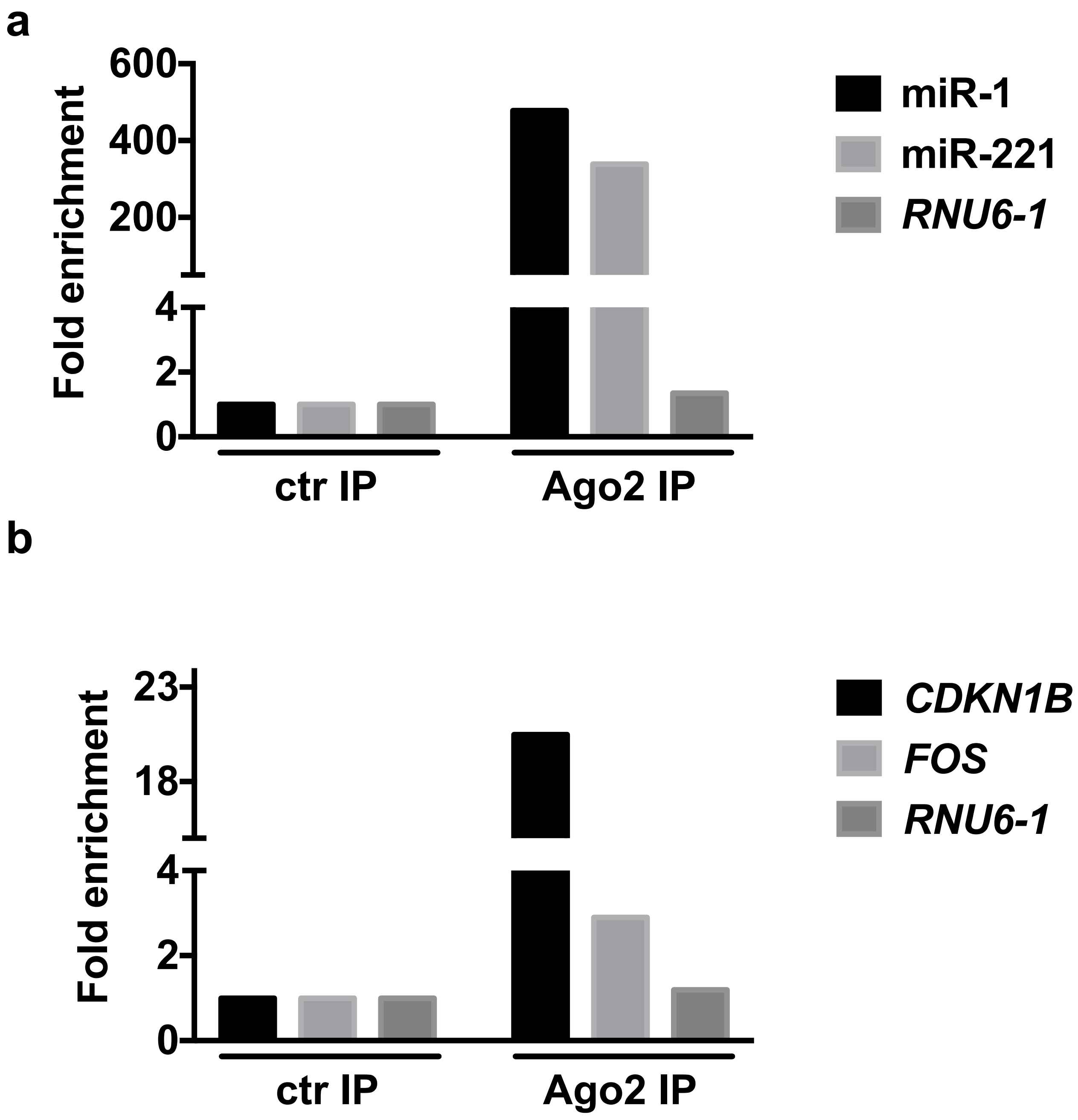

Figure S2. Quality control of RISC-associated RNAs in muscle biopsies. A fraction (10%) of RISC-associated RNAs from a control biopsy was analyzed by qPCR for miR-1, miR-221 and *U6* RNA enrichment (**a**) or for *CDKN1B, FOS* mRNAs and *U6* RNA enrichment (**b**). RN*U6-1* RNA is shown as negative control, since it is not associated to RISC. For both mRNA and microRNA analysis, differences in the amount of RNA immunoprecipitated by anti Ago2 antibodies (Ago2 IP) were calculated relative to RNA immunoprecipitated by control antibodies (ctr IP). A representative experiment is shown

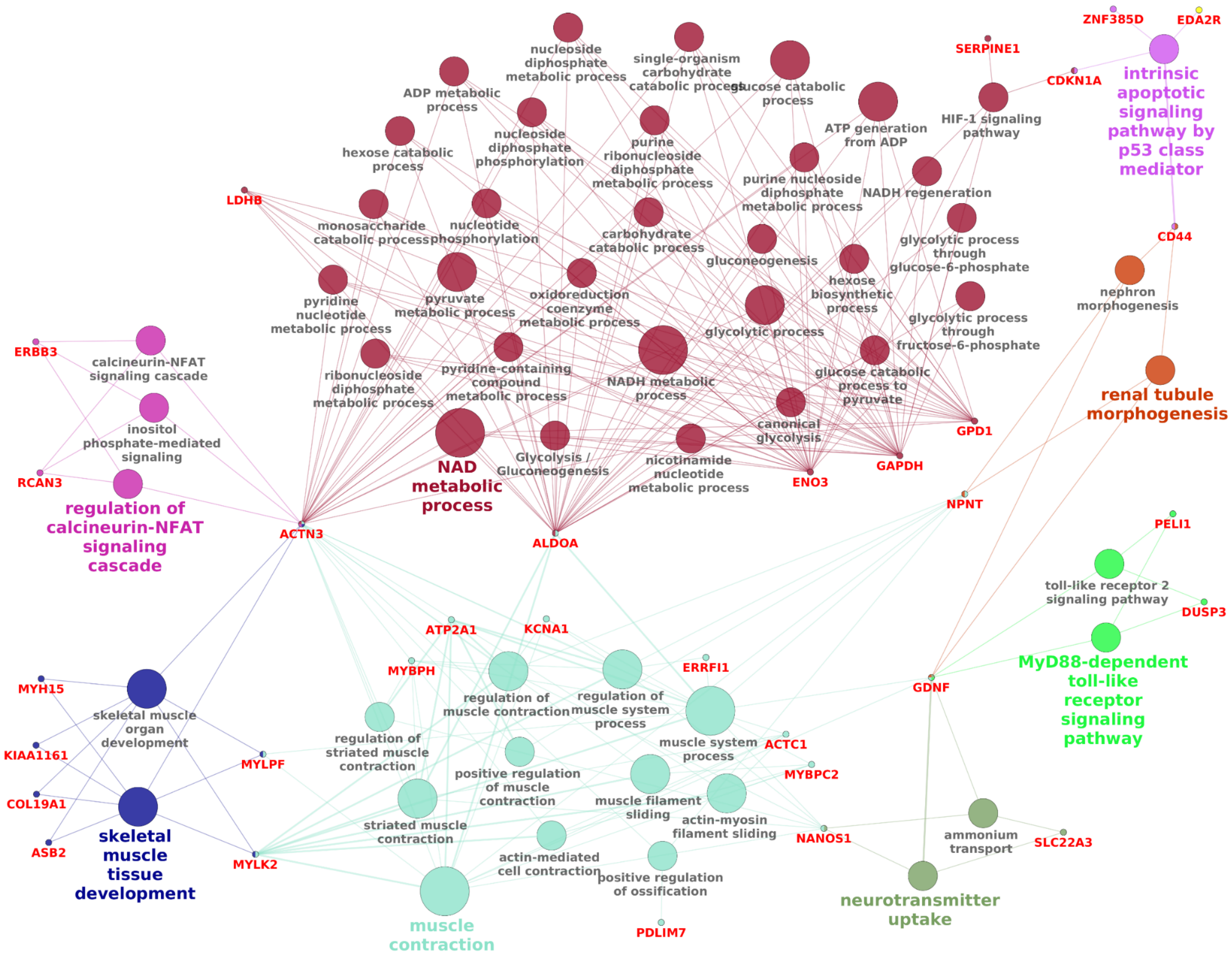

Figure S3. Gene ontology analysis of the differentially modulated mRNAs in DM1 RISC. Circles represent specific gene ontology terms (GO Biological Process) or KEGG pathways that were significantly enriched in the list of mRNAs differently associated to the RISC in DM1 patients, as shown in Table S5. Edges represent term connections within the ontology tree and colors highlight terms correlated in meaning. Term caption is coloured if the term is the most significant of the group. Gene names represent the characterizing, modulated genes that were used to start the enrichment. Edges connect genes to all the terms that contain them. Genes in red are common to two or more correlated term groups.

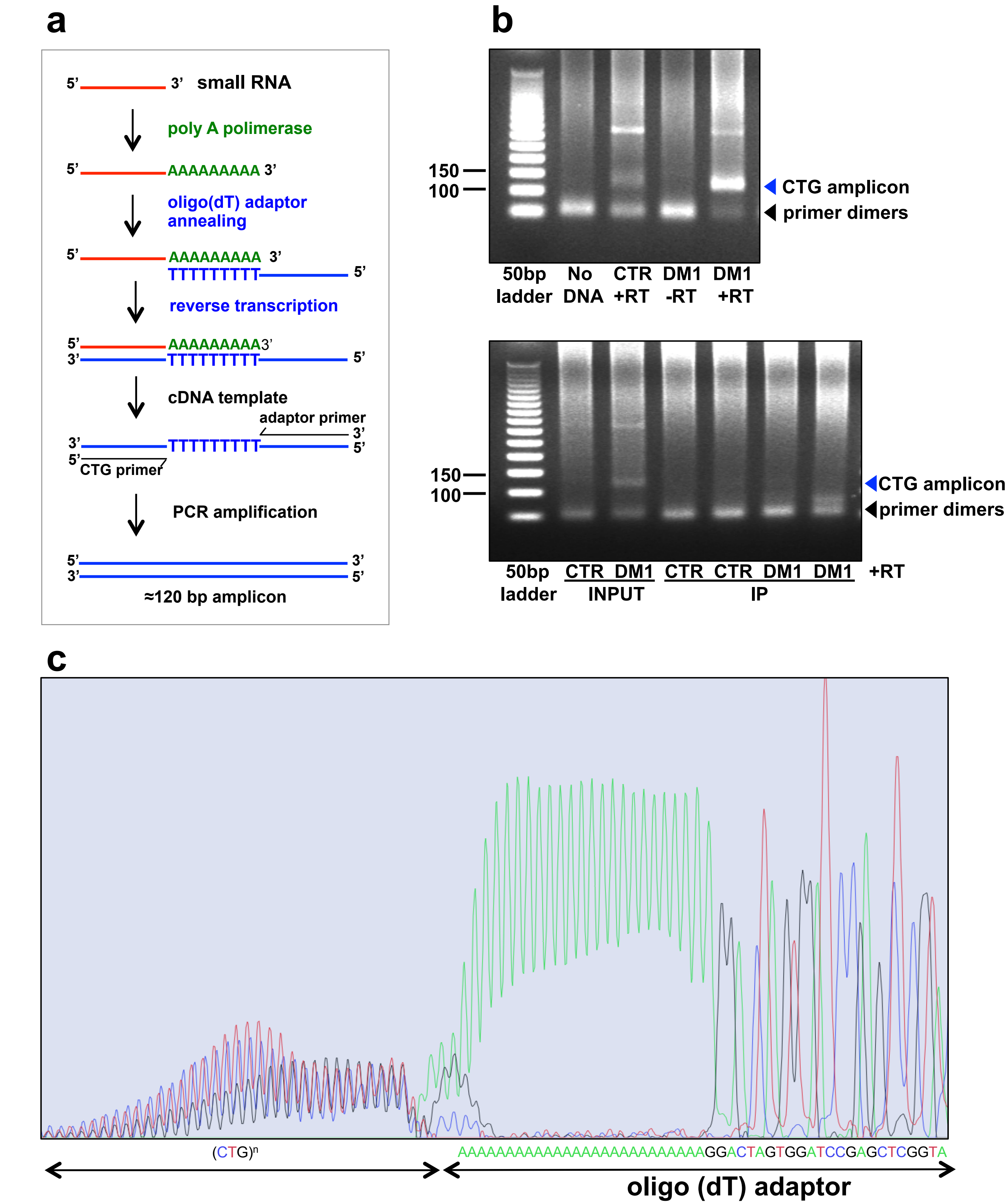

Figure S4. Amplification of CUG-containing small RNAs from DM1 muscle biopsies.
(a) Schematic representation of the CUG-RNA oligomers amplification assay. Total RNAs, including small RNAs, were polyadenylated and reverse transcribed from an oligo (dT) adaptor into cDNA. PCR using a CTG-specific forward primer and an adaptor reverse primer amplified a CTG-rich fragment of about 120nt. **(b)** CUG-containing oligomers amplification from total RNA (upper panel) and input (INPUT) or immunoprecipiatetd RISC–associated RNAs (IP, lower panel) from muscle biopsies of control individuals (CTR) and DM1 patients (DM1). CTG amplicons were detected only in total and input RNA samples from DM1 patients but not in IP samples. **(c)** Sequencing of the CTG-containing amplicon.

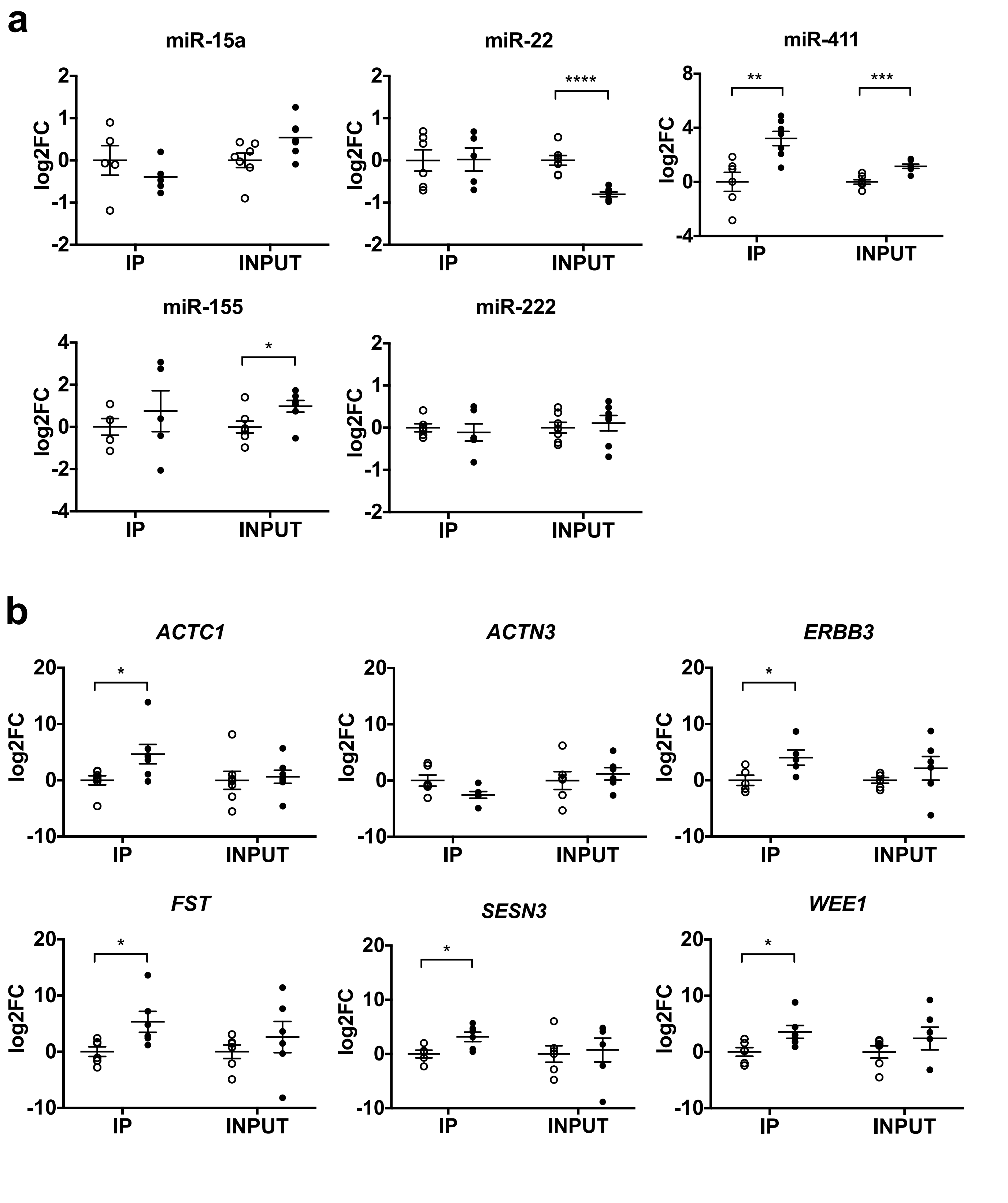

Figure S5. Validation of selected miRNAs and mRNAs differentially associated to RISC complexes of DM1 patients vs healthy subjects. Dot plots of the expression level of selected miRNAs (**a**) and mRNAs (**b**), analyzed by qPCR in RISC-IP associated RNA (IP) and INPUT RNA obtained from biopsies of DM1 patients vs healthy subjects (CTR). miRNA and mRNA levels, normalized to miR-181a and *RPL23* mRNA respectively, are indicated as -1*DDCt fold changes (log2FC). Average and error bars are also indicated *(*CTR n≥5; DM1 n≥5; *P<0.05; **P<0.01 ***P<0.001). White circles: CTR; black circles: DM1.

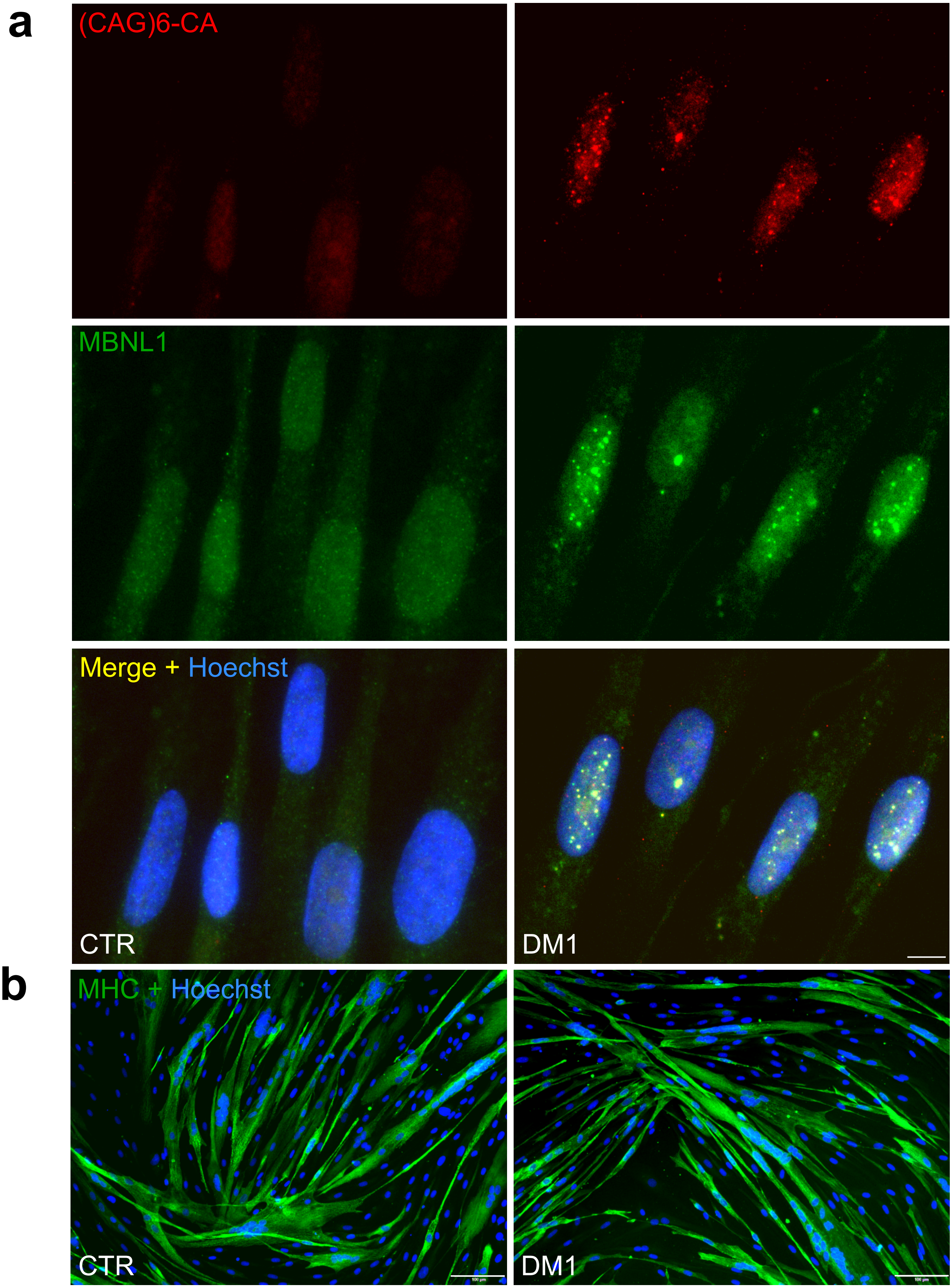

Figure S6. Characterization of DM1 myogenic cell lines. (**a**) RNA FISH analysis of CUG repeat-containing transcripts in CTR and DM1 cell lines following induction of differentiation for 2 days. Cells were double stained with Texas-Red-labeled $(CAG)_6CA$ probe and anti-MBNL1 antibody. Nuclei were counterstained with Hoechst dye. Co-localization of CUG-containing transcripts and MBNL1 protein is visible in nuclear aggregates (merge panels). Scale bar, 10 µm. (**b**) Immunofluorescence analysis of control CTR-B (CTR) and DM1-A patient-derived (DM1) cell lines allowed to differentiate for 5 days and stained with anti-MYH1 (MHC) antibody and Hoechst dye. Scale bar 100 µm

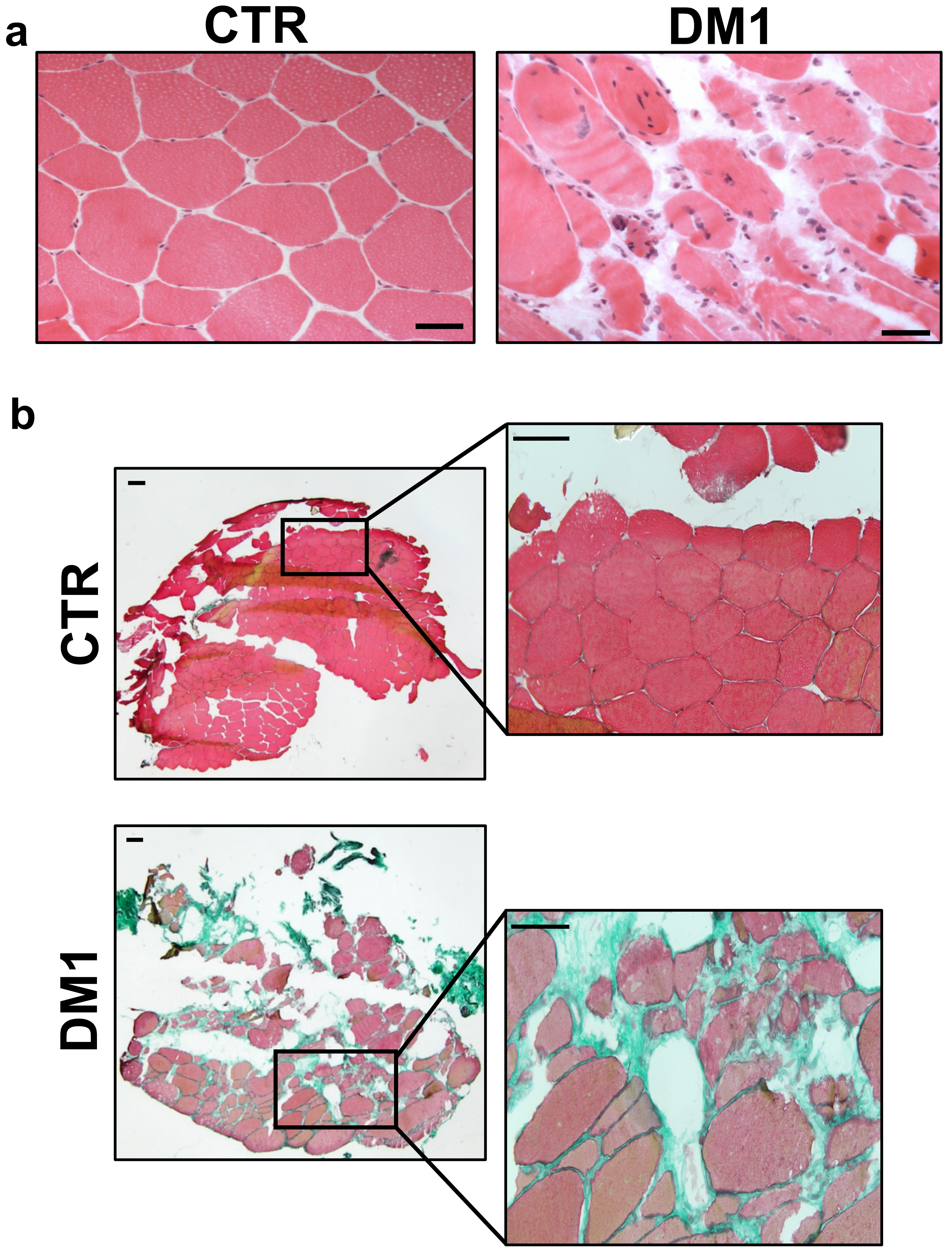

Figure S7. Pervasive fibrosis in *tibialis anterior* muscles of end-stage DM1 patients. (**a**) Representative hematoxylin and eosin stained sections shows the presence of highly damaged tissue, clearly differentiating DM1 from CTR patients. (**b**) Representative Masson's trichrome staining. The presence of fibrotic tissue is highlighted by green staining while muscle fibers are stained in red. Scale bars: 50μm

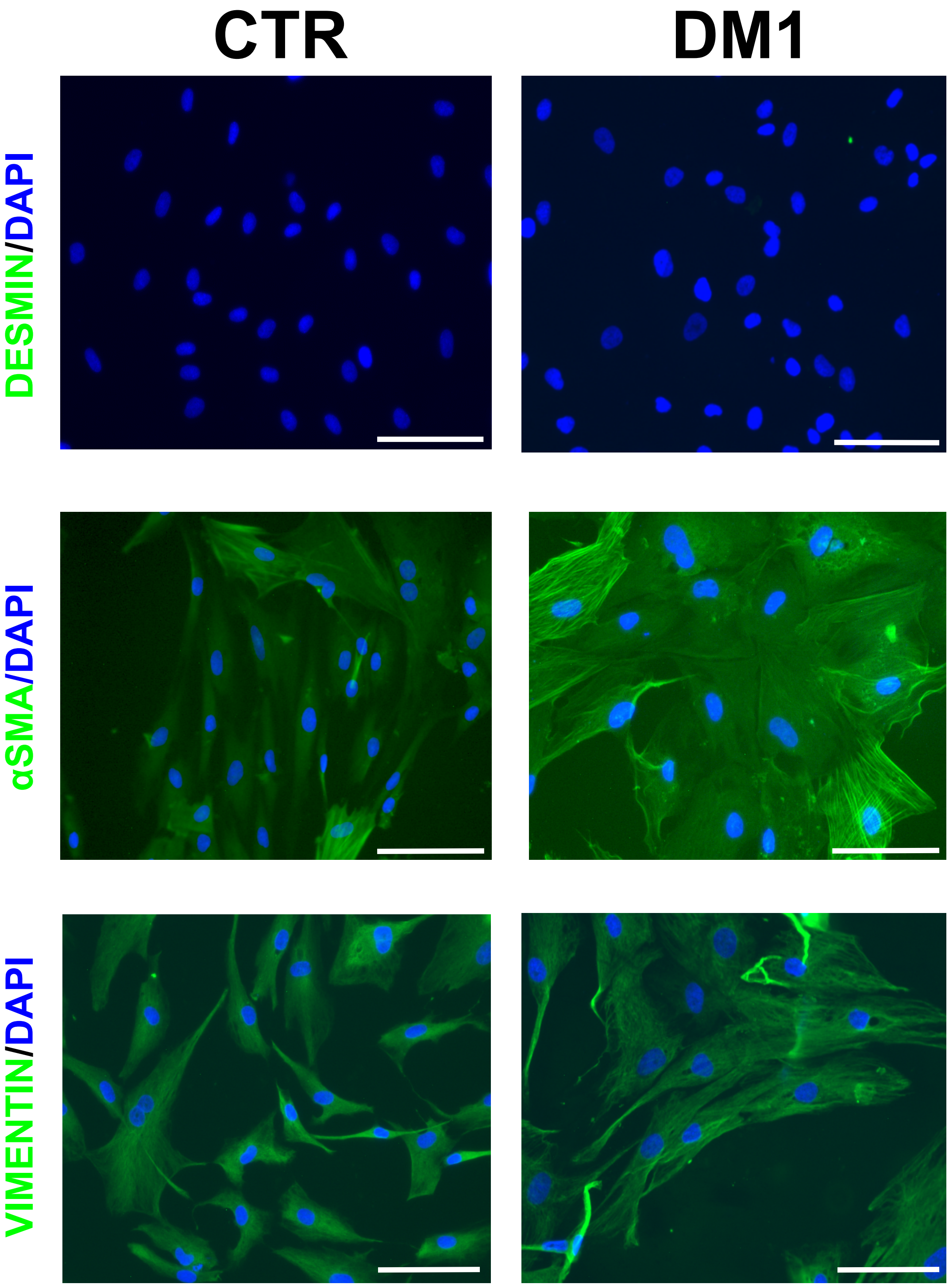

Figure S8. Characterization of skeletal muscle fibroblasts. Immunofluorescence stainings of primary fibroblasts derived from *biceps brachii* biopsies with antibodies to desmin, alpha-smooth muscle actin (alpha-SMA) and vimentin. Nuclei were counterstained with DAPI. Scale bar: 200µm

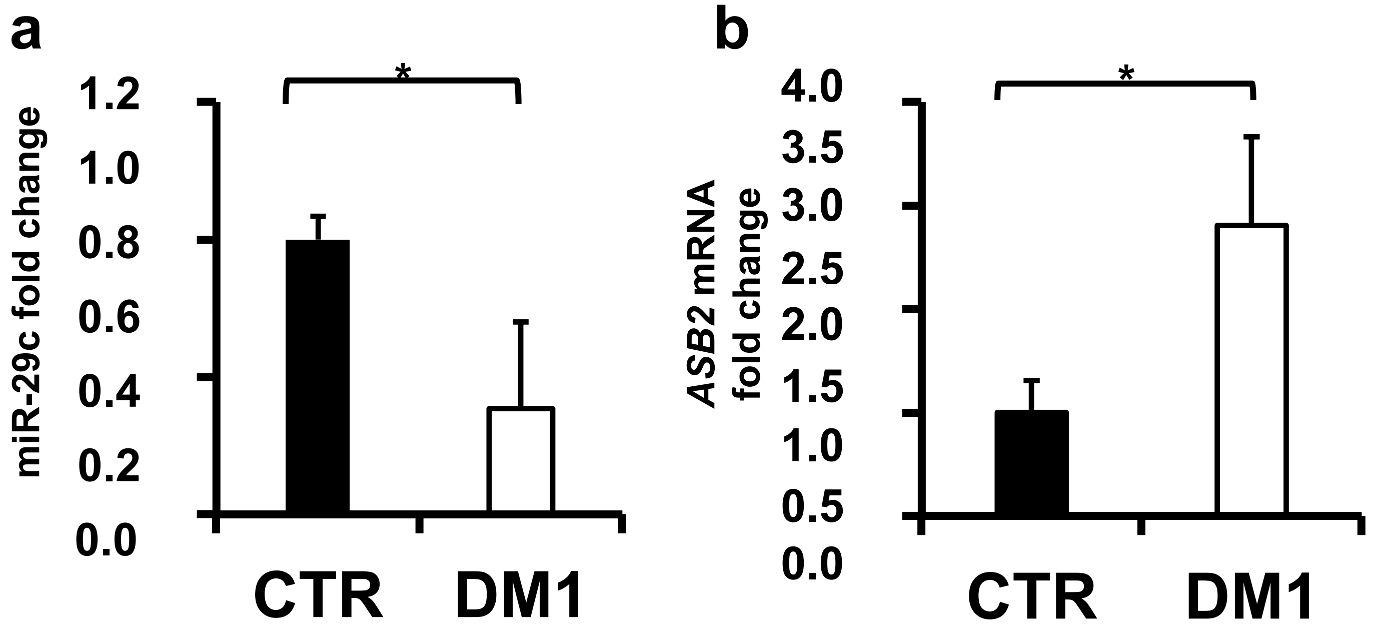

Figure S9. miR-29c and ASB2 opposite RNA modulation in primary fibroblasts of DM1 patients. Primary fibroblasts were derived from skeletal muscle biopsies of DM1 patients and controls (CTR). Total RNA was extracted and the expression of miR-29c (**a**) and its target *ASB2* (**b**) was analyzed by qPCR (DM1, n=4; CTR, n=3; *p<0.05).
